# Improvement to East African style experimental huts allows for more effective evaluation of vector control products for protection against vector-borne diseases

**DOI:** 10.1186/s13071-025-07063-9

**Published:** 2025-11-21

**Authors:** Natalie M. Portwood, Godwin Sumari, Johnson Matowo, Salum Azizi, Baltazari Manunda, Kisengwa Ezekia, Franklin W. Mosha, Louisa A. Messenger, Mark W. Rowland, Njelembo J. Mbewe

**Affiliations:** 1 Department of Disease Control, London School of Hygiene and Tropical Medicine, London, UK; 2 KCMC University-Pan African Malaria Vector Research Consortium, Moshi, Tanzania; 3 Medical Faculty, Centre for Infectious Diseases, Heidelberg University, University Hospital Heidelberg, Im Neuenheimer Feld, Heidelberg, Germany; 4 Department of Environmental and Global Health, School of Public Health, University of Nevada, Las Vegas, NV USA; 5 Parasitology and Vector Biology (PARAVEC) Laboratory, School of Public Health, University of Nevada, Las Vegas, NV USA

**Keywords:** East African experimental huts, Vector control, Mosquito behaviour, Eave baffles, Mosquito entry, Blood-feeding, Sticky traps

## Abstract

**Background:**

East African style experimental huts have been used in Tanzania since 1963 to evaluate vector control interventions such as insecticide-treated nets and indoor residual spraying. Over time, these huts have been modified to include eave baffles to minimise mosquito escape. In this study, we evaluated the impact of increasing baffle size and using netting to funnel mosquito entry into the room and prevent escape. We also explored mosquito entry behaviour using a sticky trap positioned on the hut exterior to determine if this behaviour could be leveraged for vector control.

**Methods:**

This study was conducted in Moshi, Tanzania and included two trials. In trial one, we compared the original huts with baffles with small exit holes (4 × 110 cm at the wide end nearest the eave gap and 2 × 2 cm at the narrow funnel end) to those with a larger size of baffle exit hole (20 × 120 cm at the wide end and 4 × 10 cm at the narrow end). In trial two, we compared huts with a sticky trap on the exterior wall to those without. Data analyses used logistic regression models to compare mosquito entry, blood-feeding rates and exophily, adjusting for variation between huts, cows (for blood-feeding) and days of the trial.

**Results:**

Larger eave baffles significantly increased entry of *Anopheles gambiae* Kisumu mosquitoes into huts [*p* = 0.01, adjusted odds ratio (AOR) 2.1, 95% confidence interval (CI) 1.2–3.8]. Blood-feeding rates were also significantly higher in huts with the larger baffle size compared to those with the original baffle size [*p* = 0.001, AOR 16.9 95% CI 4.9–59.0]. In trial two, 16% (95 CI 13.3–19.6) of *An. gambiae* Kisumu and 8% (95 CI: 6.3–10.5) of *Anopheles arabiensis* populations were collected on the sticky traps, significantly reducing mosquito entry into huts. With the presence of sticky traps, blood-feeding was inhibited by 12.7% for *An. arabiensis* and 32.6% for *An. gambiae* Kisumu.

**Conclusions:**

The results of this study support the use of larger baffle sizes in East African huts to capture larger numbers of mosquitoes and improve the evaluation of vector control tools. Although only a small proportion of mosquitoes were found to have had direct contact with the exterior of the hut before entry, the presence of sticky traps still reduced blood-feeding rates by limiting the entry of host-seeking mosquitoes.

**Graphical Abstract:**

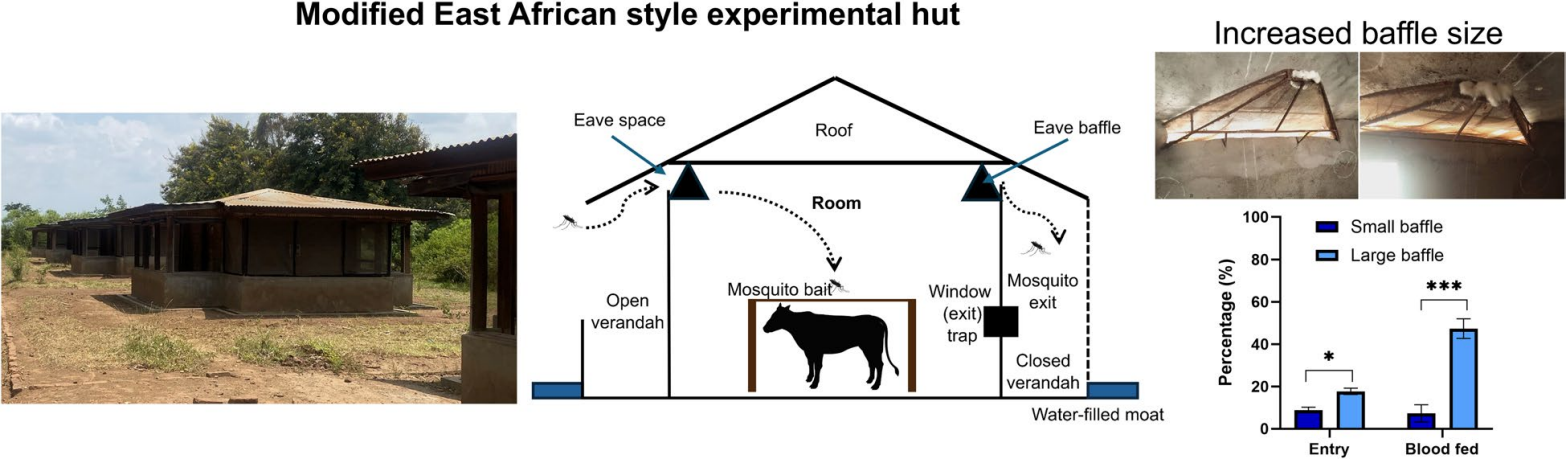

**Supplementary Information:**

The online version contains supplementary material available at 10.1186/s13071-025-07063-9.

## Background

Malaria control interventions have prevented 2.1 billion malaria cases and 11.7 million deaths between 2000 and 2022. This achievement is largely due to the scaling up of key diagnostic and treatment options and, in parallel, the employment of insecticide-based vector control interventions such as insecticide-treated nets (ITNs) and indoor residual spraying (IRS) [1]. Prior to a product being recommended by the WHO and being listed among WHO prequalified products (WHO-PQT), as required for procurement by major donors, a novel vector control product must undergo vigorous evaluation involving three phases [2]. Phase I involves laboratory testing, phase II consists of small-scale field trials in experimental huts and phase III requires large-scale field trials in communities [3].

Vector control products currently undergoing phase II evaluation include IRS and dual-active-ingredient ITNs which contain a pyrethroid insecticide in combination with a second partner chemical: a synergist, an insect growth regulator or a newly recommended pyrrole insecticide [2]. Meta-analyses have shown experimental hut studies are an important reproducible assay that capture the complex entomological efficacy of ITNs on vector populations [4]. Other such interventions include spatial repellents and house modifications, such as insecticide-treated screening of eaves and windows [5, 6]. Phase II evaluations are conducted in a controlled, semi-field setting and the efficacy of an intervention is assessed by monitoring outcomes of interest.

There are currently three different types of standardised experimental huts in sub-Saharan Africa recommended by WHO—West African style hut, East African style hut and Ifakara style huts —and their design is based on location, i.e. to replicate housing in the study area [3]. It is well understood that house design is a significant factor affecting mosquito entry and that the screening of spaces such as doors, windows and eave spaces can reduce indoor mosquito densities and thus malaria transmission [7, 8]. Therefore, standardised huts are an important tool for assessing the entomological impact of interventions on field or laboratory mosquito populations. In addition, meta-analyses of experimental hut data are increasingly used to compare interventions and parameterise mathematical models of the transmission dynamics of malaria [9–11]. Huts are designed to prevent the escape of mosquitoes, enabling the intervention to be assessed by calculating the number, mortality, blood-fed status and/or insecticide-induced changes on entering vector populations. All hut designs consist of a room for sleeping, where either human or animal bait is used for mosquito attraction, and an attached veranda trap and window traps to measure the exit rate induced by the intervention.

Current East African style verandah-trap experimental huts have been extensively used in Tanzania to evaluate several vector control interventions [12–15]. The designs of these huts evolved from simpler models that initially involved village huts with window traps added [16], and screened verandahs were later incorporated to capture mosquitoes exiting through the eave gaps [17]. These experimental huts feature two open verandahs on alternate sides and two closed verandahs, allowing mosquitoes to enter through the eave gaps, attracted by the host bait in the room for sleeping. Mosquitoes can exit through the window traps [18]. The original design of the experimental huts relied on doubling the number of mosquitoes caught in verandah traps to account for those escaped, assuming equal entry and exit rates. In recent years, further modifications to the huts have included the addition of eave baffles as a means to retain mosquitoes that enter and to produce more accurate effect size estimates [19].

Female mosquitoes rely predominantly on a combination of olfactory, visual and thermal cues to locate a vertebrate host for a blood meal [20–23]. Carbon dioxide exhaled in the breath of a host serves as a long-range attractant and behavioural activator for mosquitoes [24]. Once drawn closer by carbon dioxide, mosquitoes are further guided by additional sensory cues, such as host body odour and thermal profiles, to land on the host [25]. For malaria vectors such as *Anopheles* mosquitoes to enter a hut and feed on the host, they must enter through openings such as the eave gaps. Anopheline mosquitoes exhibit behaviour which allows them to follow odour plumes [25–27], but precisely how they navigate along such plumes through the eave gaps of a house and into the hut remains poorly understood. They may either land on the wall of the hut before entering the eave gaps or they may fly directly through the eave gaps without contacting the wall. Furthermore, the size of the eave baffle opening in East African experimental huts may affect the natural entry behaviour or limit airflow, which could reduce the effect of host-seeking cues. There is growing recognition of this potential limitation, but limited empirical evidence [28]. Therefore, in this study we evaluated whether modifying the size of the eave opening would impact mosquito entry rates and/or blood-feeding rates. As mosquito size, based on wing measurement, is considered to be an indication of fitness, possibly influencing hut entry, we also investigated whether mosquito body size influenced the likelihood of entering huts, given concerns that narrower baffles may exclude a segment of the mosquitoes seeking entry, thereby potentially introducing population-level bias in hut trial outcomes.

Understanding such behaviour is crucial for the targeted and effective application of vector control products, such as the spraying of outside walls with IRS or the innovation of novel vector control strategies, such as mosquito traps to target such vector behaviour. While previous research has primarily focused on interventions inside of huts, this study aimed to investigate the behaviour of *Anopheles* mosquitoes around eave gaps of experimental huts using sticky traps. Additionally, the study explored the impact of modifying baffle exit sizes in East African style hut designs on mosquito entry, an area that remains unexplored.

## Methods

### Study site and experimental huts

**Fig. 1:**
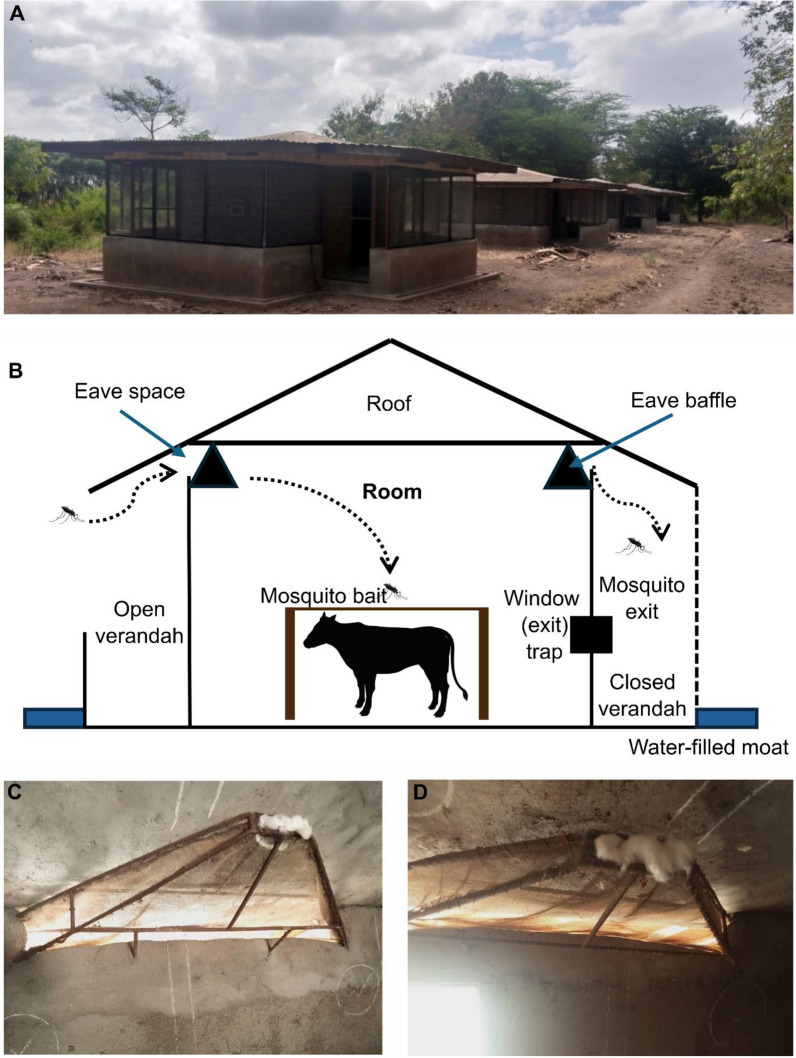
East African style huts with verandahs and baffles. **A** East African style huts used in Lower Moshi, Tanzania, modified design, constructed in 2015. **B** Schematic diagram showing the design of the hut, including the presence of eave baffles, modified from Oxborough et al. [19]. **C** Baffles with small exit holes fitted in the experimental huts (4 × 110 cm at the wide end and 2 × 2 cm at the narrow end). ** D** Baffles with large exit holes fitted in the experimental hut (20 × 120 cm at the wide end and 4 × 10 cm at the narrow end). Figure was created using BioRender.com

Two experimental hut trials were conducted at Kilimanjaro Christian Medical University College, Pan-African Malaria Vector Research Consortium (KCMC-PAMVERC) in urban Moshi, Tanzania. The studies were undertaken at the Pasua field station (03°22.764′ S, 37°20.793 E) in Lower Moshi, Tanzania. Seven East African style experimental huts are located at the site [29], all fitted with baffles in the eave gaps, with the huts found adjacent to the Lower Moshi rice irrigation system [30]. The working principle of these East African style experimental huts has been described in previous studies [19, 31, 32], as shown in Fig. 1a, b .

*Anopheles arabiensis* is the main mosquito vector in the study site, and the surrounding rice paddies are its main breeding area [33, 34]. Peak densities of *An. arabiensis* populations tend to occur in the two planting periods: June-September and November-January [29]. In this study young cows were used as bait instead of human volunteers because *An. arabiensis* has shown zoophilic feeding behaviour in Lower Moshi [34, 35]. The cows were contained in wooden enclosures of dimensions of 140 × 120 × 180 cm (width × height × length) and had to meet the following criterial: 1- or 2-year-old non-lactating calves of similar size and weight, with no insecticide treatment before or during the trial.

### Mosquito strains

In this study, wild *An. arabiensis* populations predetermined to have acquired moderate resistance to pyrethroids driven by an upregulation of cytochrome P450 mixed function oxidases were allowed to enter huts [36, 37]. Wild *An. Arabiensis* were collected as larvae and reared as an F0 generation before release. Laboratory strains, when used, consisted of the *An. gambiae* sensu stricto (*An. gambiae* s.s.) Kisumu colony, which was colonized in 1953 from Kenya. *Anopheles gambiae* Kisumu is characterised multiple times each year with respect to body weight, wing length and both phenotypic and genotypic resistance profiles. Resistance monitoring of Kisumu was regularly conducted to determine its susceptibility status prior to the study. Mosquitoes were susceptible to pyrethroids and did not possess any knockdown (*kdr*) (L1014S and L1014F) or *Ace-1* G119S mutations.

### Mosquito rearing of laboratory strains

All life stages of mosquito populations were maintained under standard insectary conditions at Harusini insectary in Moshi (25–27 °C, 80% relative humidity,). Mosquito larvae were reared in large white round bowls and fed with TetraMin (Tetra, Melle, Germany) under natural light cycles. Adult mosquitoes were kept in cages of approximate dimensions 30 × 30 × 30 cm and provided with 10% glucose ad libitum. Colony cages were maintained by regular feeding on guinea pigs for propagation of the colony.

Two trials were performed in this study. In trial 1, the effect of modifying the eave baffle size of the East African style huts was assessed; in trial 2, the effect of applying sticky traps to the outside of the experimental hut was assessed.

### Trial 1: Modification of eave baffle size of East African style huts

This trial was conducted in September 2021, using a release-recapture experimental design with laboratory strain *An. gambiae* s.s. Kisumu. The trial had two arms, a reference arm (small baffle exit hole size: 4 × 110 cm at the wide end and 2 × 2 cm at the narrow end; Fig. 1c) and a treatment arm (large baffle exit hole size: 20 × 120 cm at the wide end and 4 × 10 cm at the narrow end; Fig. 1d). Baffles were fitted onto the experimental huts, two per side (4 per hut), and evaluated. When the huts are not in use, cotton wool is used to block mosquito movement prior to the trial.

#### Release-recapture experiments

A total of 1800 unfed 2- to 5-day-old mosquitoes were released in the verandah of the huts. Since the Kisumu strain was maintained through blood-feeding on guinea-pigs, indicating zoophilic tendencies, cows were chosen as bait in the experimental hut to reflect this behaviour. The trial had two arms with each arm having a replicate, requiring four experimental huts. Treatments were rotated between two huts and tested over 9 days (Additional file 1: Figure S1). All four verandahs were screened, and mosquitoes were released at 18:00 h each day on the opposites sides of the huts that have eave baffles; simultaneously, the cotton wool was removed from the baffles. Per experimental hut on each trial day, 50 mosquitoes were released, 25 on each side, with 200 mosquitoes being released per day. Cows were rotated between huts and treatments each night to account for variation in individual attractiveness to mosquitoes or hut positional effect. Collections took place in the morning at 06:00 h, when technicians collected mosquitoes from the rooms, nets and exit and verandah traps. The following information was recorded: mosquito entry, mosquito exophily and blood-feeding rate. Mosquitoes were packed in capsules and taken for wing length measurement at the insectary at the KCMC insectary located in Moshi.

#### Wing length measurement

An ocular micrometer was placed in one eyepiece of a stereomicroscope and calibrated using a stage micrometer such that one ocular division was equivalent to 0.04 mm. For each mosquito sample, a scalpel was used to remove the left wing from the thorax. Using forceps and a dissection needle, the wing was placed on the stage of the stereomicroscope and positioned such that the alulua lined up with the “0” on the ocular micrometer. Wings were mounted in between microscope slides before measurements were taken. The number of graduations between the alula and the end of the wing where vein 3 ends were counted. The ocular graduations for each mosquito wing were recorded and multiplied by 0.04 mm to determine the wing length in millimetres.

### Trial 2: Application of sticky trap to the outside of experimental huts

**Fig. 2:**
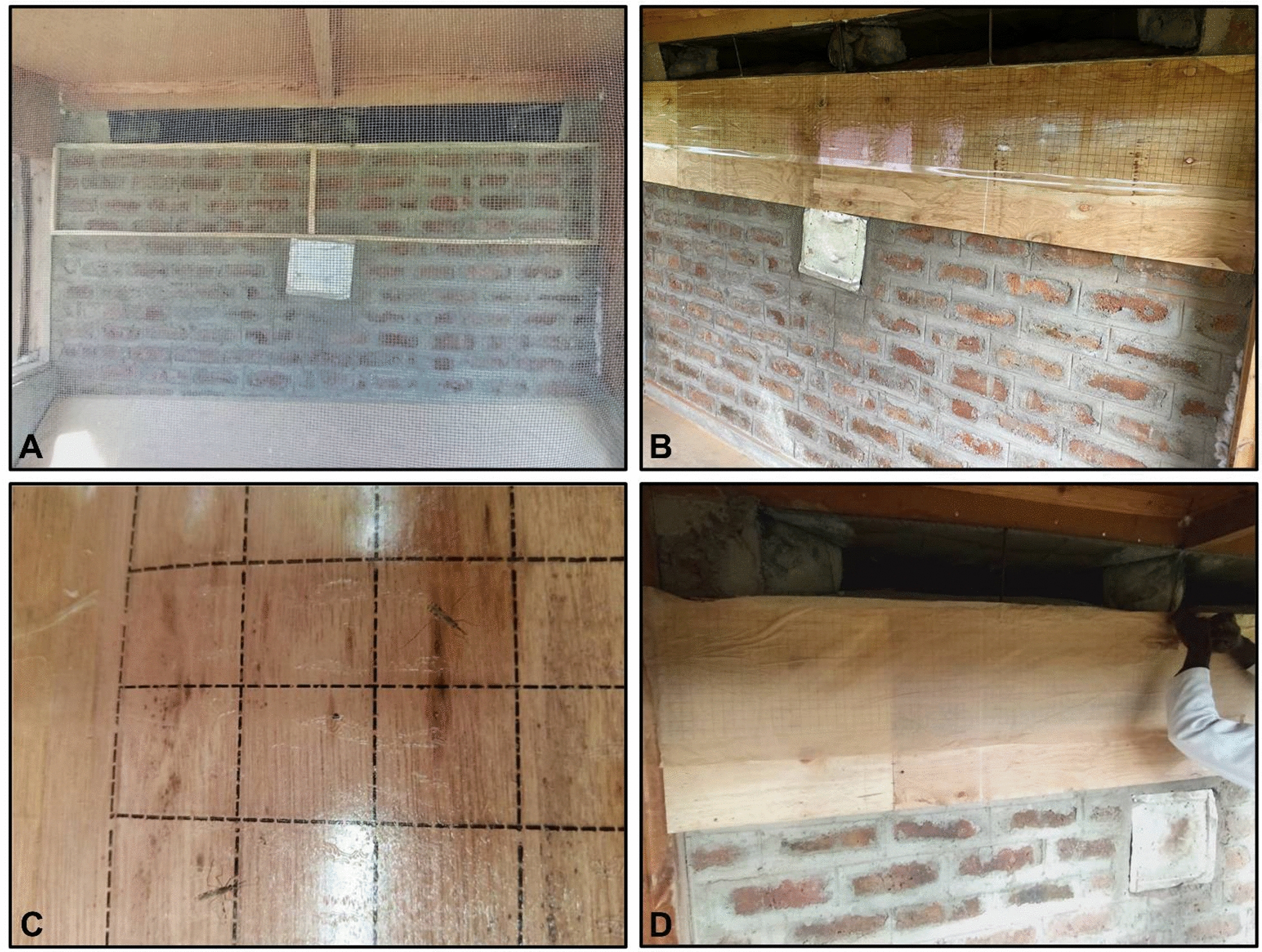
The sticky trap was fitted on the outside of experimental huts. **A** Wooden frame in which the plywood with sticky material was attached onto. **B** Sticky trap attached to the wooden frame on the outside of the experimental hut. **C** Sticky trap with two individual *Anopheles* mosquitoes caught upon contact. **D** Baking paper placed to cover the sticky material when not in use

Sticky transparent material (Luminos 4 adhesive rolls-gridded; Rentokil Initial supplies, Liverpool, UK) was stapled to a board of plywood that covered the width of the wall (approx. 2 m) and extended 30 cm down from the eave gaps (Fig. 2) as a trap for mosquitoes on the outside of the experimental hut. This set-up was designed to assess whether applying a vector control intervention to the outside of the hut would increase protection against the malaria vectors. The sticky material was applied to the sides of the huts with eave baffles, i.e. two opposite sides of the huts. A cone assay was used to make a preliminary assessment of the adhesive properties of the sticky: five *An. gambiae* Kisumu mosquitoes were exposed to the test material for 1 h, and their adherence to the surface post-exposure was observed to confirm that all mosquitoes would stick to the material upon first contact.

To assess where the mosquitoes landed on the sticky trap, a scale was marked every 10 cm downward from the eave gap to 30 cm using a black marker pen. The width of the sticky material was divided into four quadrants. The plywood was attached to the wall using a wooden bracket and secured tightly with a metal nut. When the sticky material was not in use, it was covered with baking paper.

This trial included both control and treatment arms. In the control arm, the sticky side of the material was facing inwards to avoid catching mosquitoes, but to account for any influence the pattern of the material may have on mosquito entry to the hut. For the treatment arm, the sticky side of the material was facing outwards to catch any mosquitoes which came into direct contact with the surface of the material.

#### Experimental design

A crossover experimental design was used. Two huts were used, and cows were rotated between them. Two experiments were conducted sequentially, i.e. experimental hut entrance by free-flying wild *An. arabiensis* and release-recapture experiments with unfed 2- to 5-day-old laboratory strain *An. gambiae* s.s. Kisumu mosquitoes.

#### Hut trial for sticky material

Two huts from the field station were used and cows were rotated between huts and treatments each night to account for variation in personal attractiveness to mosquitoes or positional effect of the hut. For wild free-flying *An. arabiensis,*, verandahs were opened at 18:00 h to allow mosquitoes to enter; for release-recapture experiments, 25 mosquitoes were released at 18:00 h into the verandahs, at opposite sides of the huts which had eave baffles, for a total of 50 mosquitoes per hut. The baffles were fixed on two opposite sides of each hut and not rotated to other sides during the study. A total of 1900 mosquitoes were released over 19 nights (100 mosquitoes per night). All collections were made the following morning at 06:00 h when technicians collected mosquitoes from the rooms, nets and exit and verandah traps. The entomological impact of the sticky material is expressed relative to the control group in terms of the number of mosquitoes entering the huts and the number of mosquitoes captured on the sticky trap. Secondary outcome measures included the position of the mosquitoes on the sticky material, exophily and blood-feeding rates calculated from mosquitoes caught in the room or in exit traps without the presence of the sticky trap.

### Outcome measures

The following outcomes were used, where applicable, to evaluate the efficacy of each treatment arm in the experimental hut trials:

1. Hut entry: number of mosquitoes which were collected in the experimental huts.
2. Deterrence (%): proportional reduction in the number of mosquitoes caught in treated huts relative to those caught in control huts, where *Du* is the total number of mosquitoes caught in the treated compared to control huts and *Dt* is the total number of mosquitoes caught in the treatment huts.
  $${\text{Deterrence }}\left( \% \right) = \frac{{D_{u} - D_{t} }}{{D_{u} }} \times 100$$
3. Exophily (%): exiting rates expressed as the proportion of total mosquitoes collected from the verandah and exit traps.
4. Blood-feeding (%): proportion of blood-fed mosquitoes collected in the huts or exit traps relative to the total number of mosquitoes collected.
5. Blood-feeding inhibition: proportional reduction in blood-feeding percentage in treated huts relative to that in the control huts, where *Bfu* is the proportion of blood-fed mosquitoes in untreated huts and *Bft* is the proportion of blood-fed mosquitoes in treated huts.
  $${\text{Blood feeding inhibition }}\left( \% \right) = \frac{{B_{{f_{u} }} - B_{{f_{t} }} }}{{B_{{f_{u} }} }} \times 100$$

### Statistical analyses

The entomological efficacy of each treatment was compared between baffle sizes (small and large) in trial one, and between control and treated groups in trial two, in terms of mosquito entry, blood-feeding and exophily. STATA SE version 17.0 (StateCorp, LCC, College Station, TX, USA) was used to process the study results into suitable formats to conduct analysis. Generalised linear mixed-effects models (GLMMs) were used to analyse binary outcomes (e.g. entry, blood-fed status), with a binomial distribution and logit link. Each model included treatment arm as a fixed effect and random effects for day, cow and hut to account for repeated measures and clustering. Separate models were fitted for each outcome. Additionally for trial two, to assess the proportion of mosquitoes landing on each quadrant of the sticky material and the distance from the eave gap, comparisons were done using Pearson’s chi-squared (*χ*^2^) and Fisher’s exact test. Statistical significance was determined when* p* < 0.05. All graphs were designed using GraphPad Prism 10.2.2 (GraphPad Software, San Diego, CA, USA).

## Results

### Trial 1: modification of baffle size in East African-style huts

**Fig. 3:**
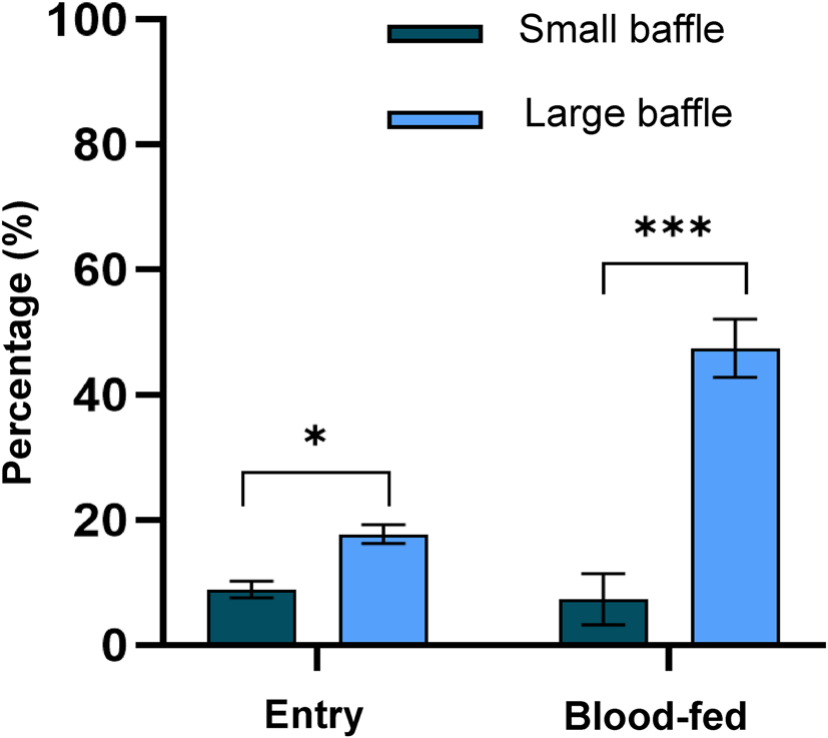
Comparison of vector entry and blood-feeding between experimental huts with different baffle sizes. Dark blue represents the baffle with small exit holes (small) and light blue represents the baffles with large exit holes (large). Error bars represent the standard error of the mean. Statistical analysis was calculated using generalised linear mixed effects models while naturally adjusting for clustering effects. Asterisks indicate significant differences between baffle sizes at *p-value < 0.05 and ***p-value < 0.001

**Fig. 4:**
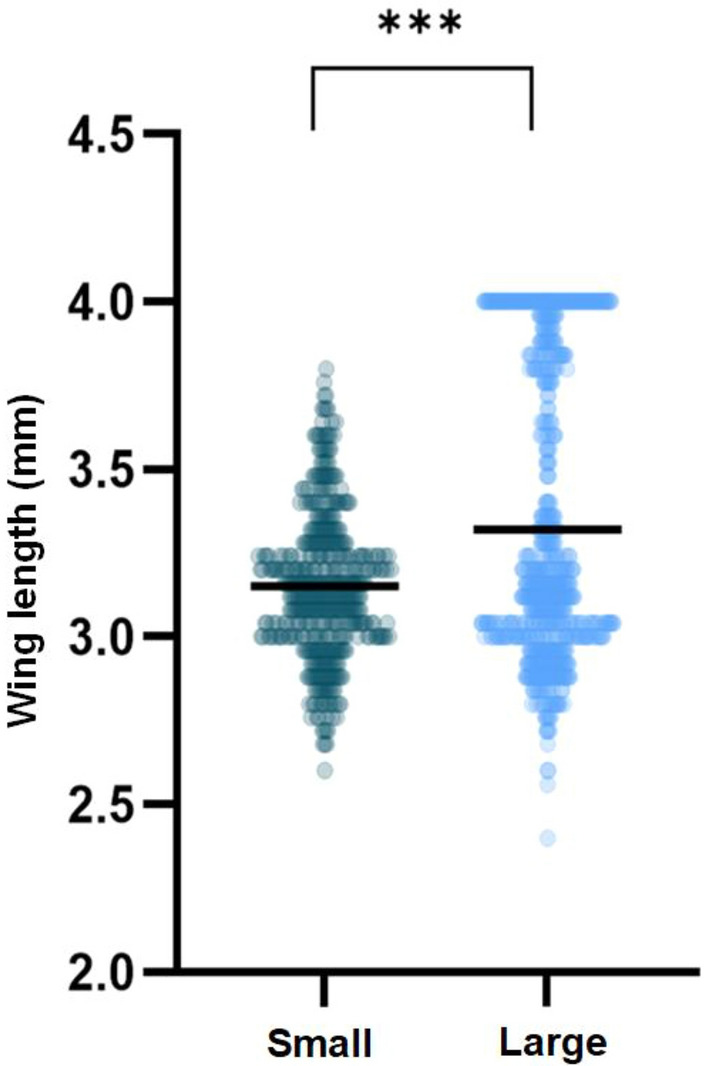
Significant increase in mosquito wing length (mm) from those collected in large baffle exit holes. Black line is the median. Statistical analysis was calculated using generalised linear mixed effects models while naturally adjusting for clustering effects. Asterisk indicate significant differences between baffle sizes at ***p-value < 0.001

A total of 1106 (61%) *Anopheles gambiae* Kisumu mosquitoes were recaptured following the release–recapture experiments over a 9-day period. The entrance rates of mosquitoes into experimental huts was significantly higher in those huts with the large baffle exit holes than in the huts with small baffle exit holes [*p* = 0.01, adjusted odds ratio (AOR) 2.1, 95% confidence interval (CI) 1.2- 3.8], as shown in Fig. 3, Table 1 and Additional file 1: Table S1. The percentage of blood-fed mosquitoes was significantly higher in the huts with the large baffle exit holes that in those with the small baffle exit holes [*p* = 0.001, AOR 16.9 95% CI 4.9–59.0] (Fig. 4).

**Table 1:** Evaluation of the efficacy of larger baffle exit holes as a modification to East African huts with *Anopheles gambiae* Kisumu populations, with outcomes adjusted for random effects due to huts and trial night

Outcomes	Small baffle exit holes	Large baffle exit holes
Total females recaptured (*n*)	463	643
Total mosquitoes entered (*n*)	41	114
% Mosquito entering (95% CI)	8.9 (6.4–11.8)*	17.1 (14.9–20.9)*
Total females that blood-fed (*n*)	3	54
% Blood-feeding mosquitoes (95% CI)	0.7 (0.1–1.9)*	8.4 (6.4–10.8)*

*CI* Confidence interval

*p-value < 0.05, indicating a significant difference between small and large baffle exit sizes

Further statistical analysis was conducted to see if there was an effect on mosquito size and baffle exit hole size. We found no association between mosquito size and hut entry or blood-feeding status (Additional file 1: Table S2). The mean wing size for mosquitoes in huts with small baffle exit holes and those with large baffle exit holes was 3.15 (95% CI 3.13–3.17) mm and 3.32 (95% CI 3.29–3.35) mm, respectively. A significant difference was found between wing size and those mosquitoes collected in huts with small and large baffle exit holes [*p *= < 0.001, AOR 1.08, 95% CI 1.06–1.1].

### Trial 2: evaluation of the application of a sticky material trap to the outside of East-African experimental huts

**Fig. 5:**
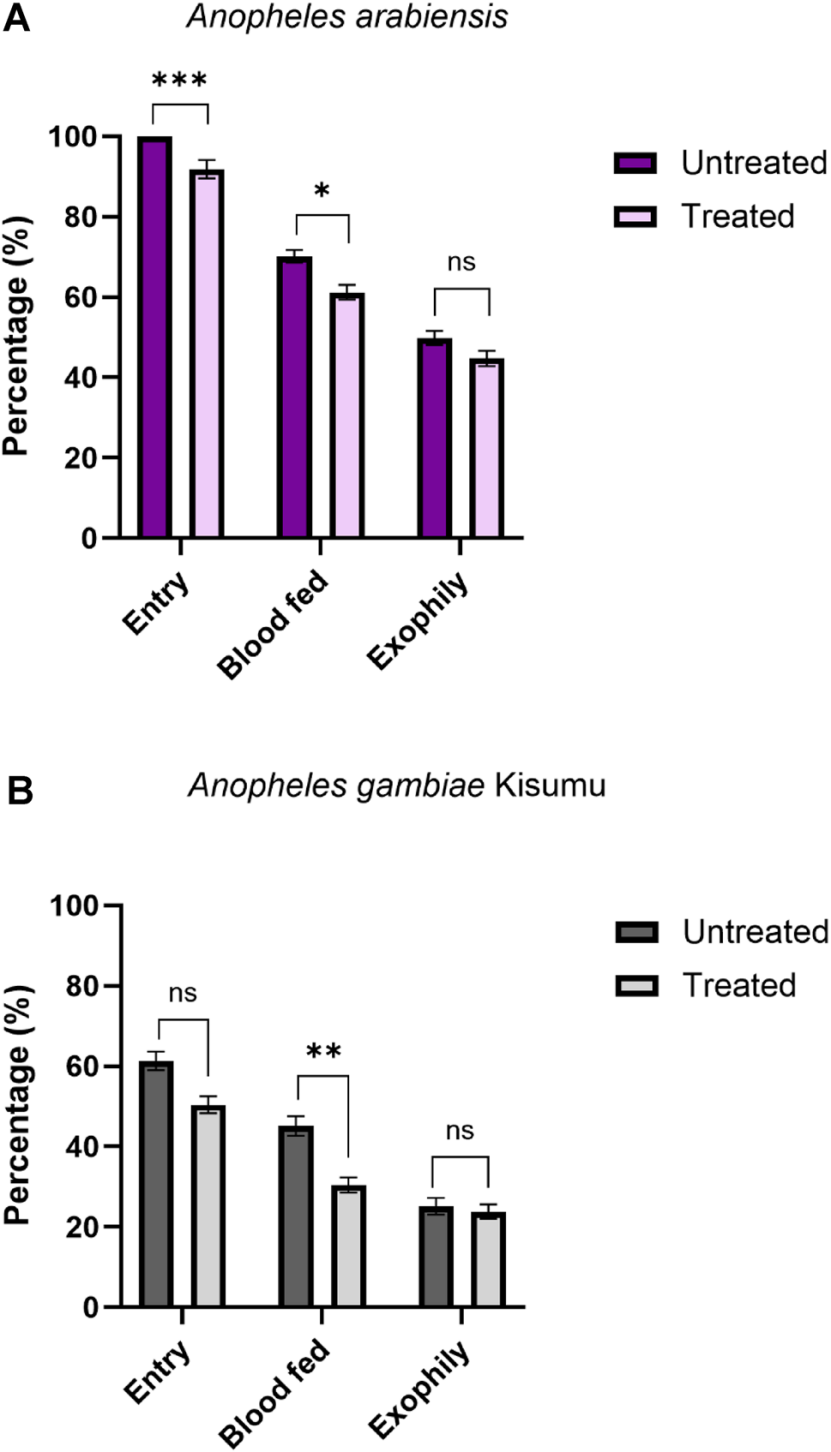
Comparison of untreated and treated (sticky material traps) groups by entrance, blood-feeding and exophily rates. **A** *Anopheles arabiensis,* **B** *Anopheles gambiae* Kisumu. Error bars represent the standard error of the mean. Statistical analysis was calculated using generalised linear mixed effects models while naturally adjusting for clustering effects. Asterisks indicate significant differences between groups at *p-value < 0.05, **p-value < 0.01 and *** *p*-value < 0.001

The preliminary cone bioassays demonstrated that the sticky material (Luminos 4 adhesive roll-gridded) possessed strong adhesive properties, with *An. gambiae* Kisumu mosquitoes adhering to the surface upon first contact, and remaining attached over the 24-h observation period (Additional file 1: Figure S2). In the experiment with wild, free-entering *An. arabiensis* populations, a total of 1483 mosquitoes were collected over 29 trap nights; in the experiments without releases with laboratory strain *An. gambiae* Kisumu, 998 (53% re-captured) mosquitoes were collected over 19 trap nights. In total, 91 (16%, 95 CI 13.3–19.6) and 58 (8%, 95 CI 6.3–10.5) *An. gambiae* Kisumu and *An. arabiensis*, respectively, were collected on the sticky material trap, thereby reducing mosquito entry into huts (Fig. 5; Table 2). For both *An. arabiensis* and *An. gambiae* Kisumu, reductions in the proportion of mosquitoes which blood-fed in the huts were reported. The presence of the sticky material inhibited blood-feeding by *An. arabiensis* and *An. gambiae* Kisumu by 12.7% and 32.6%, respectively (Table 2).

**Table 2:** Evaluation of the efficacy of a sticky material trap, against wild *Anopheles arabiensis* and colony *Anopheles gambiae* Kisumu populations, with outcomes adjusted for random effects due to cows, huts and trial night

Outcomes	*An. arabiensis*		*An. gambiae* Kisumu	
	Untreated	Treated (sticky material traps)	Untreated	Treated (sticky material traps)
Total females caught (*n*)	775	707	439	559
Total mosquitoes entered (*n*)	775	649	269	282
% Mosquito entry (95% CI)	100	91.8 (89.5–93.7)*	61.3 (56.5–65.9)	50.4 (43.0–51.1)
Deterrence (%)	–	8.2	–	17.8
Total mosquitoes exited (*n*)	386	316	110	133
% Exophily (95% CI)	49.8 (46.2–53.4)	44.7 (41.0–48.4)	25.1 (21.0–29.3)	23.8 (20.3–27.5)
Total females blood-fed (*n*)	543	433	198	170
% Blood-feeding (95% CI)	70.1 (68.0–74.5)	61.2 (57.5–64.9)*	45.1 (40.4–49.0)	30.4 (25.6–34.4)*
% Blood-feeding inhibition	–	12.7	–	32.6

*CI* Confidence interval

*p-value < 0.05, indicating a significant difference from the control group

With the presence of the sticky material trap, there was a significant reduction in the number of *An. arabiensis* entering the huts [*p* ≤ 0.001, AOR 0.06, 95% CI 0.015-0.022-1] and in blood-feeding rate [*p *= 0.02, AOR 0.6, 95% CI 0.38–0.92], but not in exophily [*p* ≤ 0.98, AOR 1.1, 95% CI 0.62–1.55] (Fig. 5; Table 2; Additional file 1: Tables S3–S5).

Similarly, for *An. gambiae* Kisumum there was a significant reduction in mosquito entry with the presence of the sticky material trap according to unadjusted odds ratios [*p* ≤ 0.005, OR 0.7, 95% CI 0.54–0.99] but not according to the adjusted odds ratios [*p *≤ 0.12, AOR 0.7, 95% CI 0.44–1.1] (Additional file 1: Table S3). Also, for *An. gambiae* Kisumum, there was a significant reduction in blood-feeding rate [*p *= 0.004, AOR 0.5, 95% CI 0.34–0.81] but no difference in exophily [*p* ≤ 0.47 AOR 0.8, 95% CI 0.49–1.38] (Fig. 5b; Additional file 1: Tables S4, S5).

Data were recorded to determine if there was a pattern to the position in which mosquitoes landed on the sticky material trap, according to distance from the eave gap and position across the house, measured in quadrants. Pearson chi-squared tests revealed that both the *An. arabiensis* and *An. gambiae* Kisumu populations showed no statistical relationship regarding the position of the mosquito on the trap (Additional file 1: Tables S6, S7).

## Discussion

East African style experimental hut studies have widely been used for the evaluation of insecticide-based vector control evaluation, including ITNs and IRS, since the 1960s. Over time, their design has evolved to enhance their accuracy [16, 17, 19]. Modifications continue to be explored, with the aim to improve their capabilities to evaluate vector control interventions at the household level. A larger collection of mosquitoes enables more detailed analyses, such as, for example, characterisation of species, resistance mechanisms and blood meal sources, and increases the statistical power for drawing conclusions. Previous work in Tanzania has shown that the addition of eave baffles reduces the proportion of mosquitoes escaping undetected through open eaves [19, 28]. In the data presented here, we have demonstrated that increasing the size of the baffle exit hole, compared to small baffle holes previously used, led to a greater number of *An. gambiae* Kisumu mosquitoes entering the huts and increased the number of mosquitoes which blood-fed.

This increase in mosquito entry and blood-feeding rate may be attributed to a change in airflow dynamics, which previous studies have shown can influence mosquito behaviour [21–23, 25, 26]. A large baffle exit hole may enhance airflow, improving the concentration and homogeneity of odour plumes. Thus, enhanced airflow through large exit holes facilitates the detection of host-emitted cues, such as carbon dioxide and other attractant molecules exhaled by the host, which in turn serve as long-range attractants and behavioural activators for mosquitoes [24, 25].

The results of this study also showed that increasing the baffle size resulted in a significant increase in the mean size of mosquitoes entering the huts. Although some bias towards whole numbers was observed in the wing length measurements, this increase may be correlated to enhanced airflow and odour dispersion, both of which facilitate higher concentrations of odour cues, easier physical entry and navigation through gaps. These findings suggest that narrower openings may selectively exclude larger mosquitoes, potentially skewing populations and affecting the interpretation of trial outcomes—similar to biases seen in trap design evaluations [38]. Based on these findings, we recommend the use of the larger-sized baffle entry hole to maximise unfed host-seeking mosquito entry and of netting that should reduce their escape. These adaptations should also funnel outdoor blood-fed mosquitoes seeking a safe refuge into the room. Further studies should explore the impact of different baffle sizes on mosquito entry and investigate how changes in airflow dynamics may influence mosquito behaviour and, consequently, improve the evaluation of vector-control interventions in East African experimental huts.

In trial two, we examined mosquito landing patterns in an attempt to understand how mosquitoes navigate odour plumes when entering experimental huts. The data revealed that the use of sticky traps led to a significant decrease in both mosquito entry and blood-feeding rates for both *An. arabiensis* and *An. gambiae* Kisumu. Although a significant decrease was observed, the majority of mosquitoes entered the huts without contacting the exterior wall, likely navigating straight into the hut without contacting the exterior wall surface. However, we cannot rule out the possibility that mosquitoes may enter from above or from the sides of the eave gaps, where sticky traps were absent. Among those mosquitoes which entered the huts, 67% of *An. arabiensis* and 60% of *An. gambiae* Kisumu subsequently blood-fed, indicating a strong host-seeking drive in the entering population. The presence of unfed mosquitoes resting on inner walls or captured in exit traps could be due to several factors, such as, for example, failed feeding attempts, insufficient time to blood-feed before collection or lack of host-seeking upon entry. Furthermore, among the mosquitoes which landed on the sticky trap, no patterns were observed in the positions in which they first contacted the trap, providing no additional insights into their navigation through odour plumes. Future studies should consider assessments with sticky materials placed in the vicinity of the eave baffles.

Despite the significant reductions observed in mosquito entry and blood-feeding rates, the question remains of whether these are substantial enough to have a large impact on malaria transmission rates as, for example, there was only a 9% and 15% reduction in blood-fed *An. arabiensis* and *An. gambiae* Kisumu mosquitoes, respectively. However, this proportion of mosquitoes landing on the outside of the hut could be exploited to provide personal protection against the malaria vector and inform rational strategies for the management of insecticide resistance. Such strategies could involve house modifications, such as the addition of a trap and/or screen and the incorporation of an insecticide, such as partial IRS or mosquito trap [39]. While selective spraying has been used for the control of *Aedes* mosquitoes in urban settings [40], its application to malaria vector control remains limited [39]. In particular, outdoor residual sprays (ORS) have proven to be successful in Malaysia, where they have been used successfully against exophilic malaria vectors, achieving sustained reductions in mortality of vector populations over several months [41]. Applying an insecticide to exterior walls may support insecticide resistance rotation programmes by enabling the use of a broader range of chemicals, thereby reducing reliance on pyrethroids, which are currently used in all ITNs, and help limit the buildup of pyrethroid resistance. Nonetheless, further research is needed to evaluate the environmental impact, cost-effectiveness and epidemiological impact of ORS in African contexts, particularly regarding non-target species and implementation feasibility.

The study has a number of limitations. It focused on only one wild strain (*An. arabiensis*) and one laboratory strain (*An. gambiae* Kisumu), so the findings may be species-specific and limited in terms of behavioural diversity. As laboratory strains like Kisumu may differ in behaviour from wild mosquitoes due to reduced genetic variation from long-term colonization [42], the current results should be interpreted with caution. While a release-recapture experimental design was used with Kisumu, it does not reflect natural entry behaviours of wild mosquitoes. The study was conducted at a single site with a limited sample size, which may restrict generalisability to other settings. Additionally, cows were used as bait instead of humans, which may influence host-seeking responses.

Further research should include other species, including *An. funestus,* another major malaria vectors vector in Africa, to better understand the behaviours of malaria vectors. Investigating mosquito behaviours following sub-lethal insecticide exposure is also critical, as these compounds can alter mosquito behaviour. Insecticides such as pyrethroids are known to alter mosquito behaviours by inducing excito-repellency and increasing exophily, which may result in altered patterns of movement through the eaves [43]. A female mosquito may encounter sub-lethal doses of insecticides from ITNs several times during her lifetime, considering she takes a blood meal every 3 days. Such exposures could potentially alter her blood-seeking behaviour and ability to navigate odour plumes. Advances in automated tracking tools, such as three-dimensional tracking systems, may soon enable detailed monitoring of mosquito behaviour surrounding experimental huts [44]. When considering house modifications to reduce mosquito biting and therefore transmission in communities by exploiting the entrance behaviour, this study supports the introduction of smaller eave gaps, screens or traps.

## Conclusions

The results of our study lead us to recommend that East African experimental huts be modified to have the large baffle exit hole size, allowing for the study of disease-transmitting mosquito behaviours in the semi-field setting and to evaluate the efficacy of indoor vector control technologies. The large baffle exit hole size captured a larger number of mosquitoes whilst preventing their escape through eave gaps, thereby enhancing the reliability of evaluations. Additionally, this study found that only a small proportion of mosquitoes came into direct contact with the exterior of the experimental hut before entry. However, the presence of the sticky traps still reduced blood-feeding rates by limiting the entry of host-seeking mosquitoes.

## Supplementary Information

**Additional file1: Figure S1.** **Study design illustration.** **Figure S2. Preliminary assessment of stickiness Luminos 4 adhesive rolls-gridded material.** **Table S1. Measures of association between treatment arms, entry and blood feeding rate.** **Table S2. Measures of association between treatments arms, wingsize data, entrance and blood feeding rate.** **Table S3.** **Measures of association between treatment arms and entry.** **Table S4. Measures of association between treatment arms and exophily.** **Table S5. Measures of association between treatment arms and blood-feeding rate.** **Table S6.** **Pearson chi-squared test and Fishers exact test to assess if there was an association landing position of mosquitoes on the sticky material panel (quarters 1,2,3 or 4).** **Table S7. Pearson chi-squared test and Fishers exact test to assess if there was an association with the landing position of mosquitoes on the sticky material panel (distance from eave gaps 0-10cm, 11-20cm or 21-30cm).**

## Data Availability

Data supporting the main conclusions of this study are included in the manuscript.

## Abbreviations

AOR: Adjusted odds ratio
GLMMS: Generalised linear mixed-effects models
IRS: Indoor residual spraying
ITN: Insecticide-treated net
WHO-PQT: WHO Prequalified Products
