# Supplementary material for: Improvement to East African style experimental huts allows for more effective evaluation of vector control products for protection against vector-borne diseases
Source: Parasit Vectors. 2025 Nov 21;18:477. doi: 10.1186/s13071-025-07063-9 (PMC12639978; doi:10.1186/s13071-025-07063-9)
Supplement: Supplementary file 1 — Additional file1: Figure S1. Study design illustration. Figure S2. Preliminary assessment of stickiness Luminos 4 adhesive rolls-gridded material. Table S1. Measures of association between treatment arms, entry and blood feeding rate. Table S2. Measures of association between treatments arms, wingsize data, entrance and blood feeding rate. Table S3. Measures of association between treatment arms and entry. Table S4. Measures of association between treatment arms and exophily. Table S5. Measures of association between treatment arms and blood-feeding rate. Table S6. Pearson chi-squared test and Fishers exact test to assess if there was an association landing position of mosquitoes on the sticky material panel (quarters 1,2,3 or 4). Table S7. Pearson chi-squared test and Fishers exact test to assess if there was an association with the landing position of mosquitoes on the sticky material panel (distance from eave gaps 0-10cm, 11-20cm or 21-30cm). [file 13071_2025_7063_MOESM1_ESM.docx]

**Supplementary Materials**

**Trial 1: Modification to eave baffle size of East African style huts**


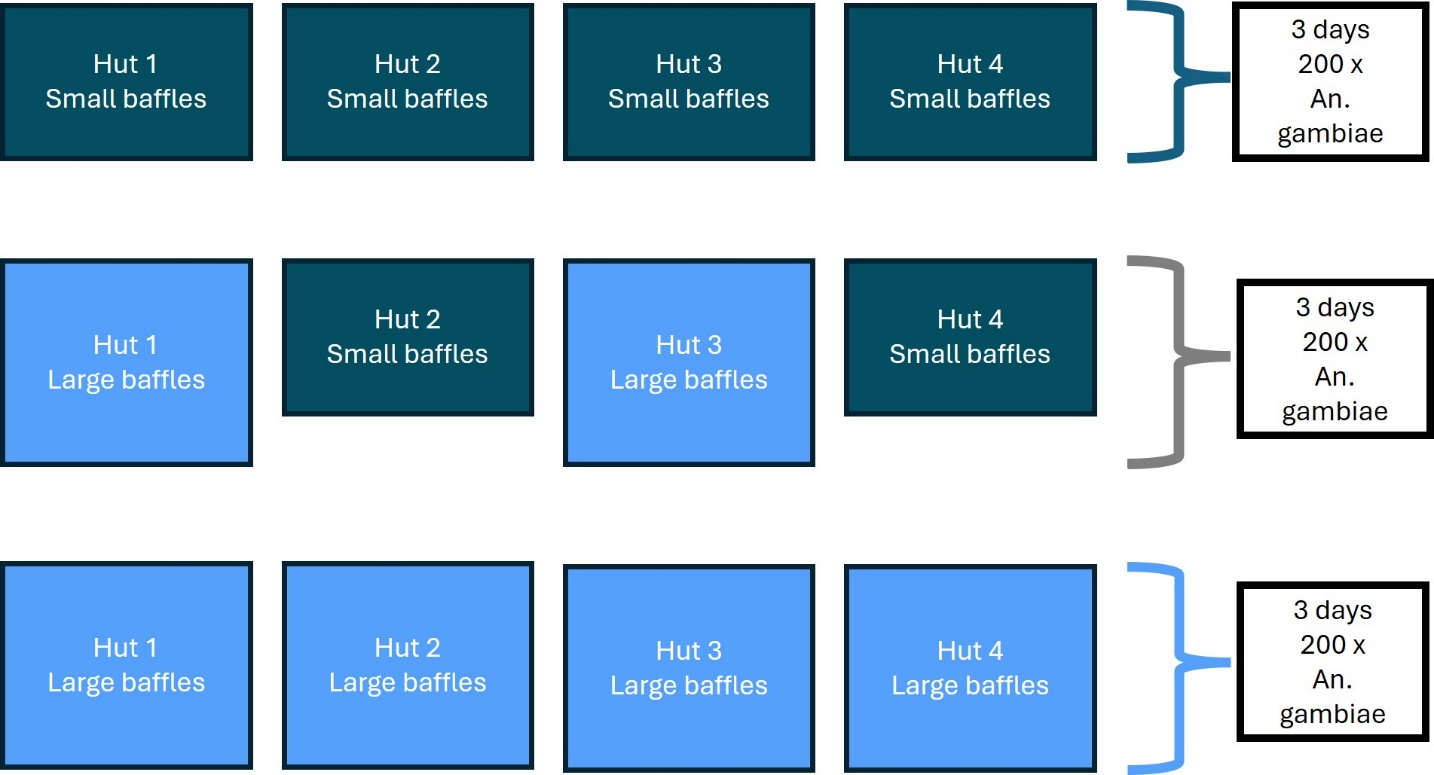


**Figure S1: Study design illustration.** There were three groups, first group - all huts had small baffles (dark blue), the second group - half the huts had large baffles (light blue) and the other half small (dark blue) and the third group – all huts had large baffles (light blue). There were three days of releases per group where 50 *An. gambiae* mosquitoes were released per hut.

**Table S1:** Measures of association between treatment arms, entry and blood feeding rate.

|  | UOR (95% CI) | p-value | AOR (95% CI) | p-value |
| --- | --- | --- | --- | --- |
| Entry | 2.2 (1.5-3.2) | < 0.001* | 2.1 (1.2-3.8) | 0.01* |
| Blood-feeding rate | 14.1 (4.4-45.2) | < 0.001* | 16.9 (4.9-59.0) | < 0.001* |

UOR is unadjusted odds ratio; AOR is adjusted odds ratio for random effects; CI is confidence interval. Asterisk is significance where p<0.05.

**Tables S2:** Measures of association between treatments arms, wingsize data, entrance and blood feeding rate.

| Reference | UOR (95% CI) | p-value | AOR (95% CI) | p-value |
| --- | --- | --- | --- | --- |
| Wingsize data | 1.06 (1.04-1.07) | < 0.001* | 1.08 (1.06 -1.1) | < 0.001* |
| Wingsize data and entry | 1.00 (0.99-1.0) | 0.68 | 1.02 (0.2-1.0) | 0.22 |
| Wingsize data and blood-feeding rate | 1.03 (1.0-1.1) | 0.046 | 1.03 (1.00-1.07) | 0.08 |

UOR is unadjusted odds ratio; AOR is adjusted odds ratio for random effects; CI is confidence interval. Asterisk is significance where p < 0.05.

**Trial 2: Evaluation of the application of a sticky material trap to the outside of East-African experimental huts**

***
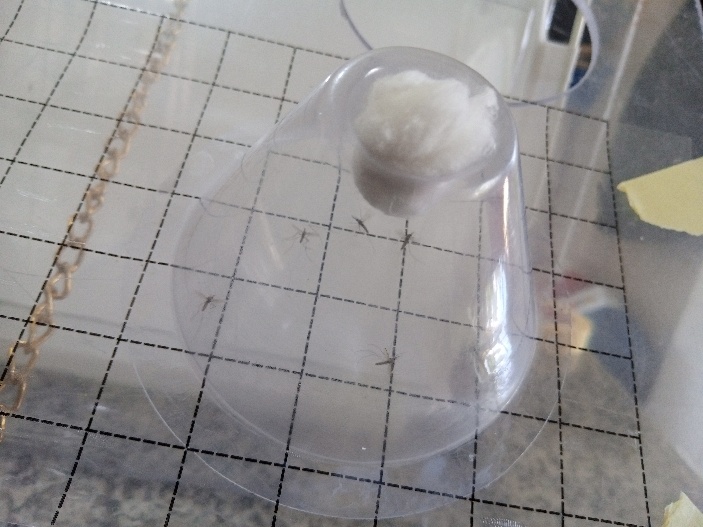
***

**Figure S2:** Preliminary assessment of stickiness Luminos 4 adhesive rolls-gridded material**.** A cone assay apparatus was used to evaluate the adhesive properties of the test material. Five *Anopheles gambiae* Kisumu mosquitoes were introduced into the cone and exposed to the material for 24 hours. Mosquito adherence to the material was observed as a measure of stickiness.

**Table S3:** Measures of association between treatment arms and entry.

| Species | UOR (95% CI) | p-value | AOR (95% CI) | p-value |
| --- | --- | --- | --- | --- |
| *An. arabiensis* | 0.08 (0.034-0.19) | < 0.001* | 0.057 (0.015-0.022) | < 0.001* |
| *An. gambiae* Kisumu | 0.70 (0.54-0.99) | 0.005 | 0.70 (0.44-1.10) | 0.12 |

UOR is unadjusted odds ratio; AOR is adjusted odds ratio for random effects; CI is confidence interval. Asterisk is significance where p<0.05.

**Table S4:** Measures of association between treatment arms and exophily.

| Species | UOR (95% CI) | p-value | AOR (95% CI) | p-value |
| --- | --- | --- | --- | --- |
| *An. arabiensis* | 0.88 (0.72-1.08) | 0.21 | 0.98 (0.62-1.55) | 0.93 |
| *An. gambiae* Kisumu | 0.79 (0.60-1.05) | 0.110 | 0.83 (0.49-1.38) | 0.47 |

UOR is unadjusted odds ratio; AOR is adjusted odds ratio for random effects; CI is confidence interval. Asterisk is significance where p<0.05.

**Table S5:** Measures of association between treatment arms and blood-feeding rate.

| Species | UOR (95% CI) | p-value | AOR (95% CI) | p-value |
| --- | --- | --- | --- | --- |
| *An. arabiensis* | 0.66 (0.53-0.82) | < 0.001* | 0.59 (0.38-0.92) | 0.02 |
| *An. gambiae* Kisumu | 0.55 (0.42-0.71) | < 0.001* | 0.53 (0.34-0.81) | 0.004 |

UOR is unadjusted odds ratio; AOR is adjusted odds ratio for random effects; CI is confidence interval. Asterisk is significance where p < 0.05.

**Table S6:** Pearson chi-squared test and Fishers exact test to assess if there was an association landing position of mosquitoes on the sticky material panel (quarters 1,2,3 or 4). Asterisk is significance where p < 0.05.

| Reference | Pearson chi-squared | p-value | Fisher’s exact |
| --- | --- | --- | --- |
| *An. gambiae* Kisumu | 174 | 0.42 | 1.0 |
| *An. arabiensis* | 174 | 0.42 | 1.0 |

**Table S7:** Pearson chi-squared test and Fishers exact test to assess if there was an association with the landing position of mosquitoes on the sticky material panel (distance from eave gaps 0-10cm, 11-20cm or 21-30cm). Asterisk is significance where p < 0.05.

| Reference | Pearson chi-squared | p-value | Fisher’s exact |
| --- | --- | --- | --- |
| *An. gambiae* Kisumu | 116 | 0.43 | 1.0 |
| *An. arabiensis* | 124 | 0.43 | 1.0 |
